# Tumor Diagnosis and Treatment Based on Stimuli‐Responsive Aggregation of Gold Nanoparticles

**DOI:** 10.1002/EXP.70006

**Published:** 2025-02-06

**Authors:** Xiaowei Chang, Huaiyu Wang, Xin Chen

**Affiliations:** ^1^ Department of Chemical Engineering, Shaanxi Key Laboratory of Energy Chemical Process Intensification, Institute of Polymer Science in Chemical Engineering, School of Chemical Engineering and Technology Xi'an Jiaotong University Xi'an China; ^2^ The First Affiliated Hospital of Xi'an Jiaotong University Xi'an China

**Keywords:** gold nanoparticles, stimuli‐responsive aggregation, tumor diagnosis and treatment

## Abstract

Gold nanomaterials have been used in the diagnosis and treatment of different tumors due to their unique physical and chemical properties. Among them, gold nanoparticles with stimuli‐responsive aggregation functions have attracted extensive attention because they can meet the unique needs of tumor diagnosis and treatment at different stages through structural changes. However, how to effectively modify gold nanoparticles to achieve structural transformation for specific stimuli, and the role of corresponding structural transformation in improving the effect of diagnosis and treatment still lack systematic summary. In this review, we comprehensively summarized the current strategies for inducing gold nanoparticles aggregation and its advances in tumor diagnosis and treatment.

## Graphical abstract

This review summarizes the current strategies for inducing gold nanoparticles aggregation and its advances in tumor diagnosis and treatment. The authors hope this review can provide a valuable reference for the further development of AuNPs clinical applications.

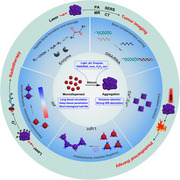

## 1 Introduction

Gold nanoparticles (AuNPs) have attracted much interest in tumor diagnosis and treatment due to their intrinsic properties such as ease of fabrication, ease of surface modification, good biocompatibility and unique optical properties [1]. For example, AuNPs can be used as tumor imaging contrast agents for photoacoustic (PA) imaging [2], surface‐enhanced Raman scattering (SERS) imaging [3], magnetic resonance (MR) imaging [4] and computed tomography (CT) imaging [5], and they can also be used for photothermal therapy (PTT) [6], radiotherapy [7], drug delivery [8] and nano‐enzymes (glucose oxidase, GOD; peroxidase, POD) [9]. Many previous reviews summarized the superiority of AuNPs and their application in biomedical applications from different perspectives. For instance, Tamarkin et al. first summarized AuNPs as tumor‐targeted drug delivery vehicles [10], Astruc et al. comprehensively reported the application of AuNPs in nanomedicine, including preparation, diagnosis and therapy [11]; Dykman et al. summed up the in vivo biodistribution and toxicity of AuNPs [12]; Rotello et al. focused on chemical and biological sensing [13] and Allen et al. reviewed AuNPs in radiation therapy from a mechanistic perspective [14].

The tumor diagnosis and treatment of different stages need different states of AuNPs. Monodisperse small‐size AuNPs are required to achieve long blood circulation and deep tissue penetration during the tumor accumulation stage [15]. Aggregated large‐size AuNPs are required to prolong tumor retention time to improve the efficiency of diagnosis and treatment [16]. Therefore, many studies have focused on the construction of intelligent AuNPs with stimuli‐responsive aggregation function to simultaneously meet the different needs of tumor diagnosis and treatment at different stages. However, how to effectively fabricate AuNPs to achieve stimuli‐responsive aggregation, and the role of corresponding aggregation in improving the effect of diagnosis and treatment still lack of systematic summary.

**SCHEME 1:**
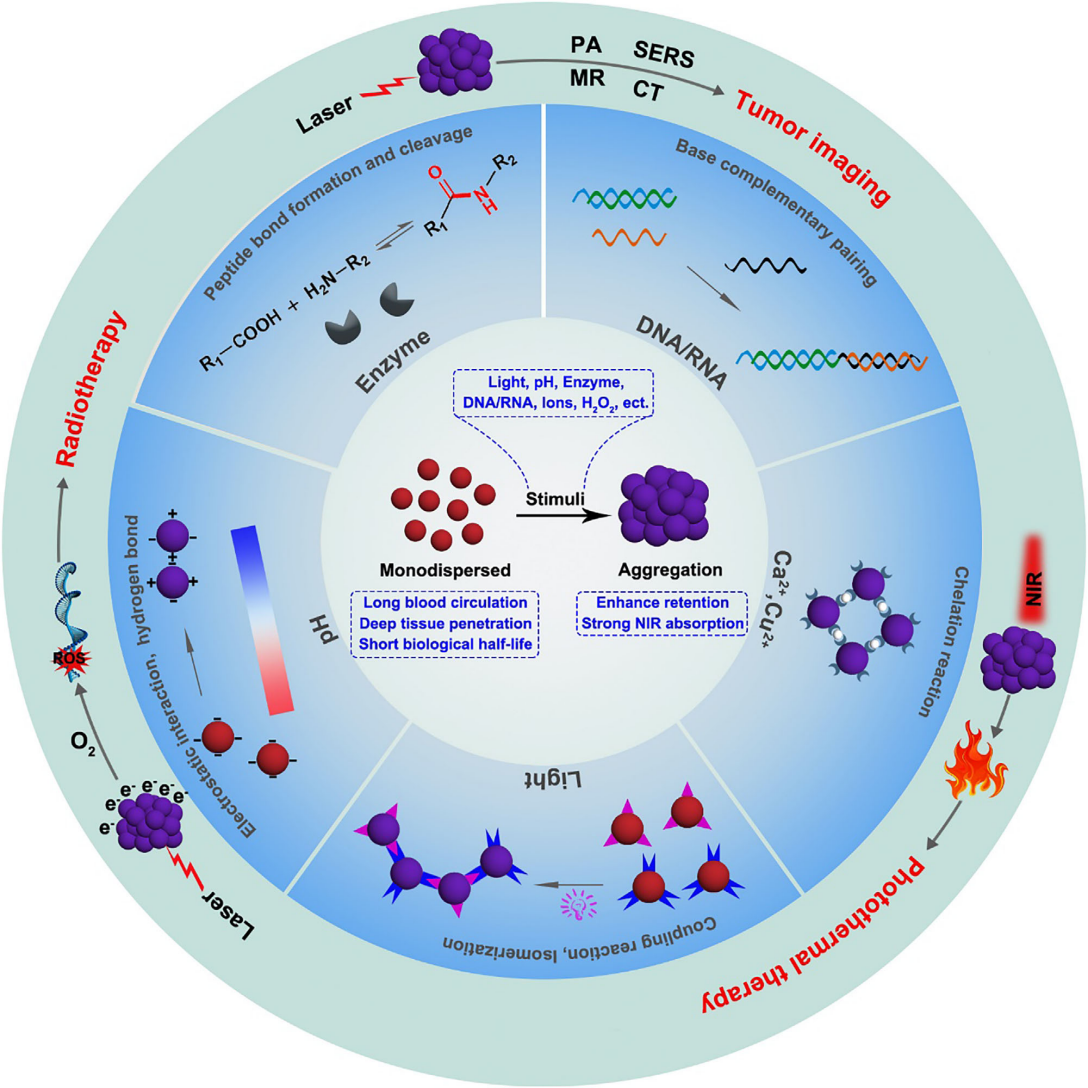
Schematic illustration of the stimuli‐responsive AuNPs aggregation for tumor diagnosis and treatment.

Therefore, different from other reviews, we summarized the current research progress in the development of stimuli‐responsive AuNPs aggregation strategies for tumor diagnosis and treatment (Scheme 1). First, we briefly discussed the reasons for designing stimuli‐responsive AuNPs aggregation strategy. Then, the function of stimuli‐responsive aggregation of AuNPs was summarized. Finally, the applications of stimuli‐responsive aggregation of AuNPs in tumor diagnosis and therapy were comprehensively summarized. This review may have important implications for the further development of AuNPs clinical applications.

## 2 Design Reasons for Stimuli‐Responsive Aggregation of AuNPs

AuNPs have the unique optical property of localized surface plasmon resonance (LSPR) in near‐infrared (NIR) regions [17], which enables them for various biomedical imaging modes such as PA, SERS, MR and CT imaging [18], as well as enables them for PTT and radiotherapy via generate local photothermal and cytotoxic reactive oxygen species (ROS) upon NIR irradiation [19]. These unique properties are closely related to its size [20]. Small‐size AuNPs (<30 nm) are more suitable for the accumulation stage of the tumor because of their deep tissue penetration, long blood circulation time and short biological half‐life [21]. In addition, small‐size AuNPs are less toxic than large‐size AuNPs (>200 nm). Studies have proved that small‐size AuNPs can eventually be cleared from the body through the liver and kidneys, rather than accumulating widely in various cells, tissues and organs [22]. However, only large‐size AuNPs aggregates have an excellent NIR absorption that is an important factor for applications of tumor imaging, PTT and radiotherapy. In addition, AuNPs aggregates inhibit efflux and prolong the retention time, which is more suitable for the treatment and diagnosis stage. Unfortunately, they are easily captured by the reticuloendothelial system, resulting in unsatisfactory tumor accumulation and unexpected toxicity [23]. Therefore, efforts have been made in recent years to manipulate the size of AuNPs during different stages of tumor diagnosis and therapy via stimuli‐responsive AuNPs aggregation strategy, which can not only maximize the accumulation and retention within the tumor, but also transfer the LSPR to the NIR region for better therapy.

## 3 Function of Stimuli‐Responsive Aggregation of AuNPs

### 3.1 Stimuli‐Responsive AuNPs Aggregation Induced LSPR Red‐Shifting

One of the unique features of AuNPs is their ability to absorb light due to LSPR, which comes from the collective oscillation of the conduction electrons across the nanoparticle because of the resonant excitation by the incoming photons [24]. The LSPR is especially influenced by the size of AuNPs. Along with AuNPs aggregation, the LSPR wavelength can red‐shifting from the ultraviolet–visible range to the NIR range, and change the solution color from wine red to purple due to the interparticle plasmon coupling [25]. This phenomenon has made them an attractive candidate for colorimetric sensors [26]. In addition, the strong NIR light absorption of AuNPs aggregates and subsequent non‐radiative energy dissipation allows for the application of PTT and PA imaging [27]. The LSPR of AuNPs aggregates can also lead to surface‐enhanced Raman scattering for SERS imaging [3c].

**FIGURE 1:**
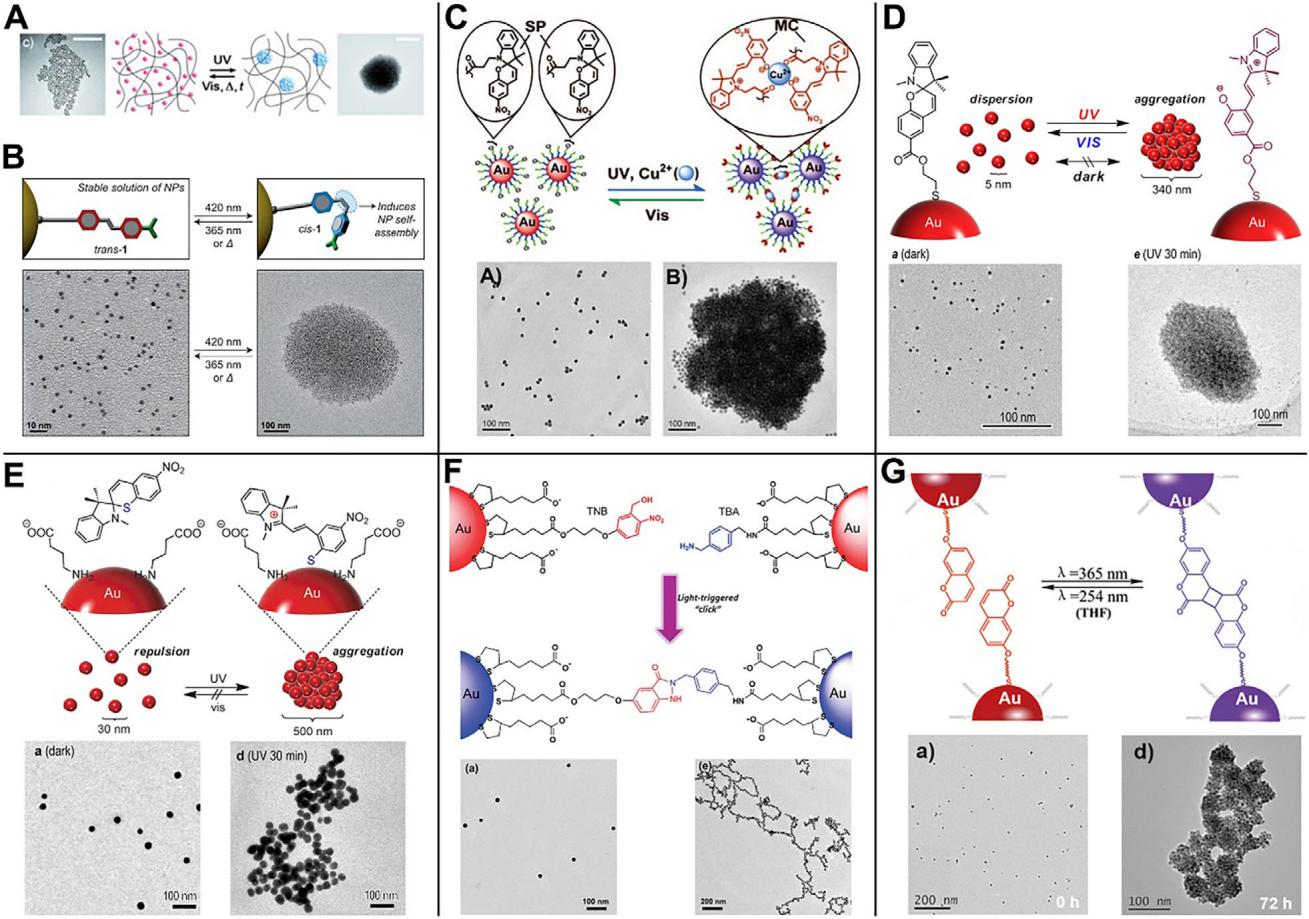
Light‐responsive aggregation strategy of AuNPs. (A) Isomerization of azobenzene. Reproduced with permission [29]. Copyright 2009, Wiley. (B) Isomerization of dimethylaminoized azobenzene. Reproduced with permission [30]. Copyright 2015, Wiley. (C) Isomerization of spiropyran and copper coordination. Reproduced with permission [31]. Copyright 2011, Wiley. (D) Isomerization of spiropyran and electrostatic interaction. Reproduced with permission [32]. Copyright 2014, American Chemical Society. (E) Isomerization of spirothiopyran and electrostatic interaction. Reproduced with permission [33]. Copyright 2013, Wiley. (F) Coupling reaction between o‐nitrobenzyl alcohol and benzylamine. Reproduced with permission [34]. Copyright 2011, Royal Society of Chemistry. (G) Dimerization of coumarin. Reproduced with permission [35]. Copyright 2016, Wiley.

Light has the advantages of spatiotemporal control and has been widely used for stimuli‐responsive induction of AuNPs aggregation for inducing LSPR red‐shifting [28]. The light‐dependent AuNPs aggregation is implemented based on light‐triggered isomerization or coupling reaction. Klajn et al. first modified azobenzene molecules onto the surface of AuNPs and achieved reversible AuNPs aggregation via light‐responsive isomerization of azobenzene (Figure 1A) [29]. Under ultraviolet (UV) irradiation, trans‐azobenzene isomerized to cis‐azobenzene, which further induced AuNPs aggregation through dipole–dipole interactions. The cis‐azobenzene was in a metastable state, which was re‐isomerized to trans‐azobenzene in the absence of UV irradiation, resulting in the AuNPs aggregates disintegrating spontaneously. Subsequently, Klajn et al. introduced dimethylamino in the para‐position of azobenzene and designed a blue/UV responsive reversible aggregation system of AuNPs (Figure 1B) [30]. Under blue light irradiation, the configuration of the dimethylaminoized azobenzene was reversed from trans to cis, which further induced AuNPs aggregation. However, under UV irradiation, it turned to trans‐azobenzene again, resulting in the redispersion of the AuNPs aggregates.

Spiropyran is another important class of light‐responsive organic molecules, which exists in non‐planar, closed form under dark or visible light conditions. Under UV irradiation, the C─O bond of spiropyran broke and isomerize to a flat, open mercyanine form [36]. Based on this property of spiropyran, a series of light‐responsive AuNPs aggregation systems had been designed. Jiang et al. modified spiropyran onto the surface of AuNPs to achieve light dependent AuNPs aggregation (Figure 1C) [31]. The C─O bond of spiropyran was broken under UV irradiation to expose OH group, which cooperated with the pre‐modified carbonyl group via specifically capturing copper ions and causing AuNPs aggregation. Upon visible light irradiation, the ring‐opening spiropyran reclosed the loop, so that the AuNPs aggregates returned to a dispersed state. Subsequently, Shiraishi et al. designed spiropyran‐modified AuNPs [32]. Under UV irradiation, the ring was opened to increase the dipole of spiropyran, which further induced AuNPs aggregation through electrostatic interaction (Figure 1D). Similarly, AuNPs aggregates dissociated into a dispersion state under visible light irradiation. Shiraishi et al. also developed an AuNPs system with irreversible aggregation [33]. Instead of directly modified onto AuNPs with spirothiopyran, they added them to a solution of 4‐aminobutyric acid modified AuNPs (Figure 1E). Under UV irradiation, the C─S bond in spirothiopyran broke to expose SH group, which in situ modified to AuNPs, resulting in a change in the surface charge and further inducing the AuNPs aggregation by electrostatic interaction.

The light‐dependent AuNPs aggregation could also be achieved by a light‐triggered coupling reaction. Zhao et al. modified o‐nitrobenzyl alcohol and benzylamine onto two kinds of AuNPs respectively, which occurred coupling reaction under UV irradiation and further induced aggregation (Figure 1F) [34]. Subsequently, Zhan et al. fabricated coumarin‐modified AuNPs (Figure 1G) [35]. Under 365 nm laser irradiation, coumarins underwent a coupling reaction to induce AuNPs aggregation. However, under 254 nm laser irradiation, the aggregates were newly dissociated into monodisperse, so as to achieve reversible AuNPs aggregation.

**FIGURE 2:**
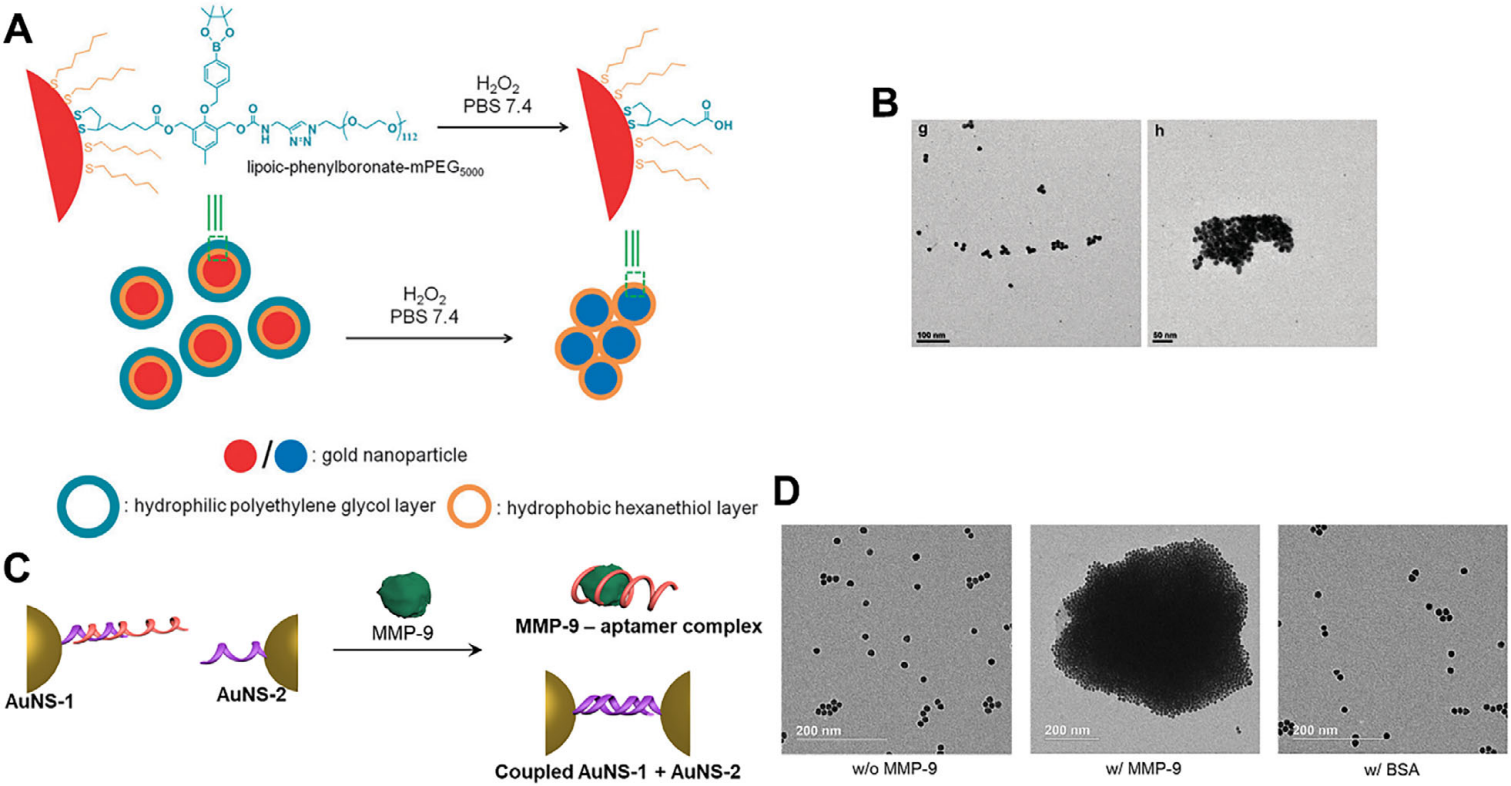
H_2_O_2_ and DNA‐responsive aggregation strategy of AuNPs. (A,B) H_2_O_2_‐responsive AuNPs aggregation. Reproduced with permission [37]. Copyright 2016, Royal Society of Chemistry. (C,D) DNA‐responsive AuNPs aggregation. Reproduced with permission [38]. Copyright 2022, Elsevier.

In addition, through the oxidation of H_2_O_2_, the hydrophilicity of AuNPs changed and hydrophobic interaction could induce AuNPs aggregation, resulting in the LSPR red‐shifting for detecting H_2_O_2_. Zhao et al. reported a H_2_O_2_‐responsive AuNPs realized hydrophobic‐to‐hydrophilic conversion in the presence of H_2_O_2_ and further induced AuNPs aggregation (Figure 2A) [37]. This AuNPs were co‐modified with two compounds: 1‐hexanethiol and lipoic‐phenylboronate‐mPEG_5000_. 1‐Hexanethiol was a short‐length hydrophobic compound that generated a hydrophobic layer around AuNPs (yellow layer). Lipoic‐phenylboronate‐mPEG5000 was a long‐length hydrophilic compound that formed a hydrophilic layer in the periphery of AuNPs. Although the lipoyl and phenylboronate fragments were hydrophobic, the long PEG chains provided good hydrophilic and space steric hindrance to prevent AuNPs aggregation in H_2_O solutions. The intermediate phenylborate unit acted as a H2O2‐sensitive structure and broke in the presence of H_2_O_2_, resulting in the PEG chains being separated and left from the AuNPs. The separation of PEG reduced the water stability of AuNPs and finally caused AuNPs aggregation via hydrophobic interaction. Direct evidence was provided by TEM in Figure 2B, which observed AuNPs aggregates after the addition of H_2_O_2_.

Through DNA complementary base pairing, AuNPs could also be self‐assembled into large‐size aggregates for red‐shifting the LSPR. Emelianov et al. developed a smart AuNPs system, which consisted of AuNS‐1 and AuNS‐2 (Figure 2C) [38]. The AuNS‐1 was modified with matrix metalloproteinase‐9 (MMP‐9) aptamer and its partial complementary strands (comp‐1). The AuNS‐2 was modified with a single‐strand fully matched complementary sequence of comp‐1 (comp‐2). In the absence of MMP‐9, the complementary pairing of comp‐2 and comp‐1 was prevented by the prebound MMP‐9 aptamer, resulting in a monodisperse state of the AuNPs. Once MMP‐9 was added, the MMP‐9 aptamer preferentially combined with MMP‐9 rather than comp‐1 to generate the MMP‐9 aptamer mixture. The MMP‐9 aptamer was released from AuNS‐1, allowing complementary pairing of comp‐2 on AuNS‐2 and comp‐1 on AuNS‐1 and further triggering AuNPs aggregation. The results showed that in the presence of MMP‐9, AuNPs aggregates were produced. In contrast, the size of AuNPs did not change without MMP‐9 or treated with BSA (Figure 2D). In addition, other stimuli such as peptides [39] and salts [40] could also achieve responsive aggregation of AuNPs for red‐shifting the LSPR. This unique LSPR property was the foundation for biological applications.

### 3.2 Stimuli‐Responsive AuNPs Aggregation Induced High X‐Ray Absorption

As one of the metals, AuNPs have X‐ray absorption property, which can absorb X‐rays and emit photoelectrons and other secondary electrons. These electrons can ionize DNA molecules directly and break the DNA strand and base [41]. Moreover, they can react with water in tissues to generate cytotoxic free radicals and further bind to DNA, causing DNA damage for radiotherapy [14]. In addition to radiotherapy, the X‐ray absorption property of AuNPs makes it possible to be used as an X‐ray CT imaging agent for tumor diagnosis. However, small‐size AuNPs have a limited X‐ray absorption coefficient, resulting in insufficient efficiency in tumor treatment and diagnosis. Therefore, a stimuli‐responsive aggregation strategy can on‐demand increase the size of AuNPs to improve the X‐ray absorption coefficient and further enhance the efficiency of tumor diagnosis and treatment.

### 3.3 Stimuli‐Responsive AuNPs Aggregation Induced Long Tumor Accumulation

The size of AuNPs greatly influences the blood circulation, biodistribution and tumor accumulation [42]. After intravenously injected into the body, the small‐size AuNPs are more likely to escape from the clearance by the mononuclear phagocytic system than large‐size AuNPs [43]. So, the small‐size AuNPs have longer blood circulation time and are easier to effectively accumulate in tumor tissue through the enhanced permeability and penetration (EPR) effect. However, after entering the tumor sites, the small‐size AuNPs can be pumped back into the bloodstream by the high interstitial fluid pressure of the tumor, resulting in a short tumor accumulation time [44]. While the large‐size AuNPs are more capable of accumulation in tumor than the small‐size. Therefore, the stimuli‐responsive AuNPs can realize better blood circulation and accumulation for meeting the unique needs of tumor diagnosis and treatment at different stages, so long as they change their sizes at the appropriate time under specific stimuli.

## 4 Stimuli‐Responsive AuNPs Aggregation for Tumor Diagnosis

Tumor diagnosis is essential for individualized cancer therapy. Therefore, based on the stimuli‐responsive AuNPs aggregation strategy, a variety of imaging techniques including PA imaging, SERS imaging, MR imaging and CT imaging have been used to achieve accurate diagnosis of tumors (Table 1).

**TABLE 1:** Summary of stimuli‐responsive AuNPs aggregation for tumor diagnosis.

Imaging type	Mechanism	Reference
PA imaging	LSPR	[45–25, 49, 50, 51]
SERS imaging	LSPR caused SERS	[52]
MR imaging	Gd^3+^‐DTPA modified on AuNPs	[53]
CT imaging	high X‐ray absorption coefficient	[54]

### 4.1 PA Imaging

PA imaging is a non‐invasive and non‐ionizing imaging method that utilizes thermoelastic expansion induced by light absorption and shows huge potential for clinical imaging applications of cancer [55]. During imaging, tumor tissue is irradiated with a non‐ionizing light, and the light energy is absorbed by endogenous components (such as hemoglobin) or exogenous contrast agents, which can be converted into images by instrument analysis for tumor diagnosis [56]. The stimuli‐responsive AuNPs have unique LSPR in the NIR region, which plays a vital role in PA imaging [57]. AuNPs aggregates can absorb light energy and convert it into heat energy under NIR irradiation, resulting in a local temperature increase. Due to the principle of thermal expansion and cold contraction, the heated tissue will undergo instant thermal expansion, leading to pressure changes and acoustic vibration. These photoacoustic signals are picked up by an ultrasound detector and further processed to produce a PA image of tumor tissue.

**FIGURE 3:**
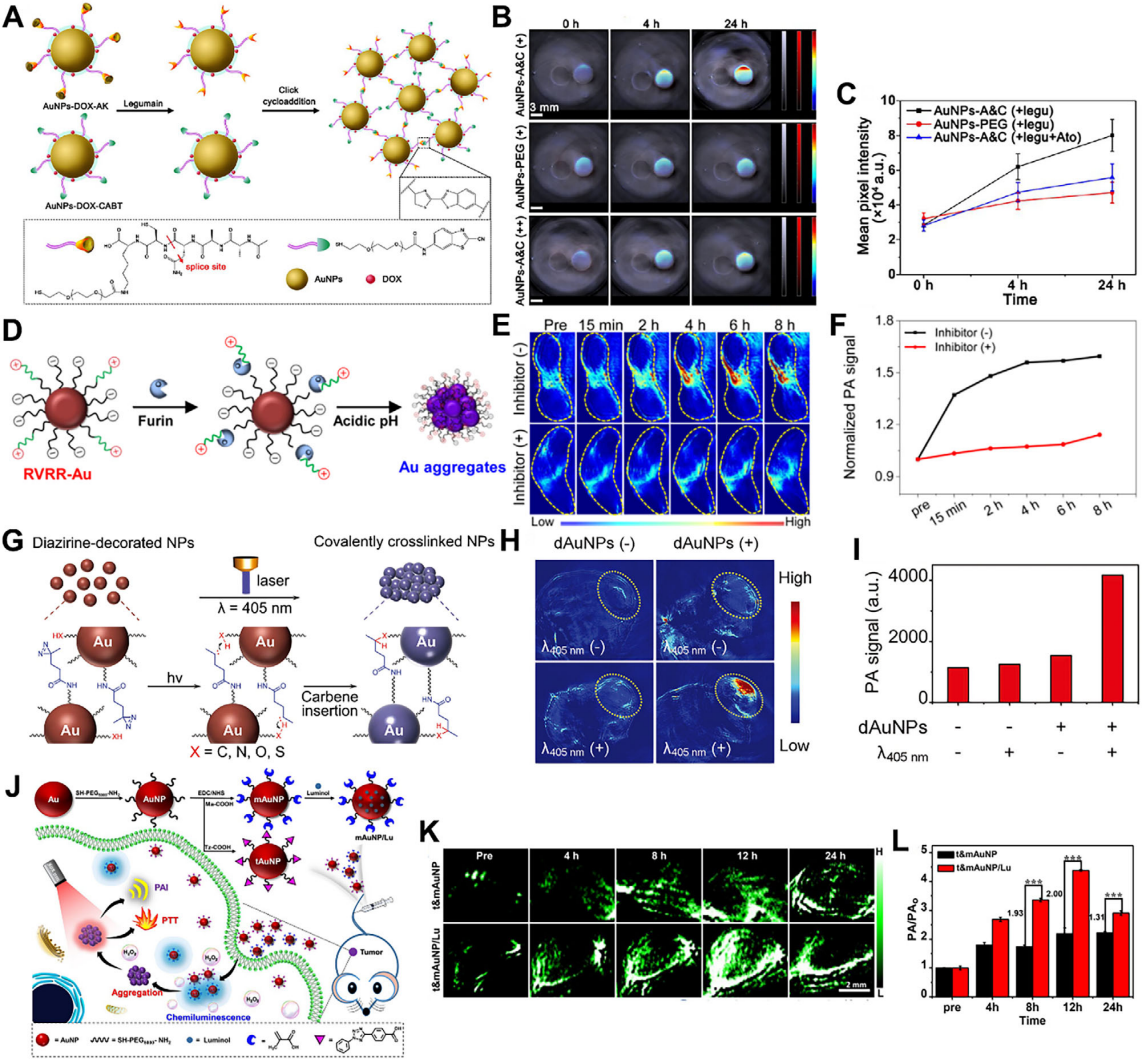
Stimuli‐responsive AuNPs aggregation for PA imaging. (A) Schematic illustration of legumain‐induced aggregation of AuNPs‐DOX‐A&C. Reproduced with permission [45]. Copyright 2016, American Chemical Society. (B,C) PA images and mean pixel intensity of AuNPs‐A&C incubated with legumain in the absence and presence of atorvastatin, AuNPs‐PEG used as a control group. Reproduced with permission [45]. Copyright 2016, American Chemical Society. (D) Schematic illustration of furin and pH synergistically induced aggregation of Au‐RRVR. Reproduced with permission [46]. Copyright 2021, American Chemical Society. (E,F) PA images and the corresponding normalized PA signal of the tumors after treatment by Au‐RRVR, the mice were pretreated with or without the furin inhibitor. Reproduced with permission [46]. Copyright 2021, American Chemical Society. (G) Schematic illustration of light‐triggered aggregation of dAuNPs. Reproduced with permission [47]. Copyright 2017, Wiley. (H,I) PA images and quantified PA signal of the tumors after treatment by dAuNPs with or without *λ* 405 nm irradiation. Reproduced with permission [47]. Copyright 2017, Wiley. (J) Schematic illustration of chemiluminescence‐induced aggregation of t&mAuNP/Lu for enhanced PAI. Reproduced with permission [48]. Copyright 2021, Wiley. (K,L) PA images and quantified PA signals of the tumors after treatment by t&mAuNP or t&mAuNP/Lu at different times. Reproduced with permission [48]. Copyright 2021, Wiley.

Gao et al. developed Ala‐Ala‐AsnCys‐Lys modified AuNPs (AuNPs‐AK) and 2‐cyano‐6‐aminobenzothiazole modified AuNPs (AuNPs‐CABT), and used them to label brain tumors via PA imaging [45]. The AuNPs‐AK could hydrolytically expose the 1,2‐thiolamino groups in the presence of legumain, which further induced a click cycloaddition reaction with the cyano group on AuNPs‐CABT, and further caused AuNPs aggregation (Figure 3A). The results indicated that AuNPs‐A&C could selectively accumulate and aggregate in the glioma site maintaining the strong PA signal, enabling accurate diagnosis of glioma. However, AuNPs‐PEG, AuNPs‐AK or AuNPs‐CABT alone were not able to cause AuNPs aggregation, resulting in a weak PA signal (Figure 3B,C).

Subsequently, Gao et al. designed a double‐responsive AuNPs (Au‐RRVR) that is able to be synergistically activated by overexpressed furin enzyme and acidic in the tumor microenvironment to induce AuNPs aggregation within tumors for enhanced PA signal (Figure 3D) [46]. After application, positively charged RVRR could be broken by furin enzyme and caused the charge of AuNPs converted to zwitterionic, which further led to electrostatic aggregation of AuNPs in acidic pH, resulting in prolonged tumor retention and enhanced PA signal. The results showed that the PA intensity at the tumor site of the experimental group was significantly strengthened with time, and reached the peak after 8 h, which was 1.6 times stronger than the initial background. In contrast, the PA signal was weak in the control group (Figure 3E,F). Gao et al. also designed a light‐responsive AuNPs aggregation strategy for PA imaging of tumor [47]. This smart dAuNPs were modified with diazirine group, which transformed into carbene under 405 nm irradiation (Figure 3G). After that, the formed reactive carbene could generate covalent bonds with ligands of adjacent AuNPs via C─C, C─H, O─H and X─H (X = heteroatom) insertions, resulting in AuNPs aggregation and further leading to a strong PA signal for tumor diagnosis (Figure 3H,I).

Considering the poor tissue penetration of external light sources, Shi et al. developed an endogenous light‐triggered aggregation strategy for PA imaging [48]. They fabricated two kinds of AuNPs (tAuNP and mAuNP) through modification of 2,5‐diphenyltetrazole and methacrylic acid onto the surface of AuNPs, respectively. Then, luminol adsorbed on mAuNPs to obtain self‐luminous mAuNP/Lu nanoparticles that were able to generate intense chemiluminescence by reaction with H_2_O_2_, which further triggered cycloaddition of tAuNP and mAuNP causing AuNPs aggregation and led to enhanced retention for PA imaging (Figure 3J). As depicted in Figure 3K,L, compared with the control group after treatment by t&mAuNP, the PA intensity at the tumor site after treatment by t&mAuNP/Lu increased significantly over time. In addition, several pH [49], HOCl [25], salt [50] and glutathione [51] responsive AuNPs aggregation systems also had been developed to enhance PA signals for tumor diagnosis.

### 4.2 SERS Imaging

**FIGURE 4:**
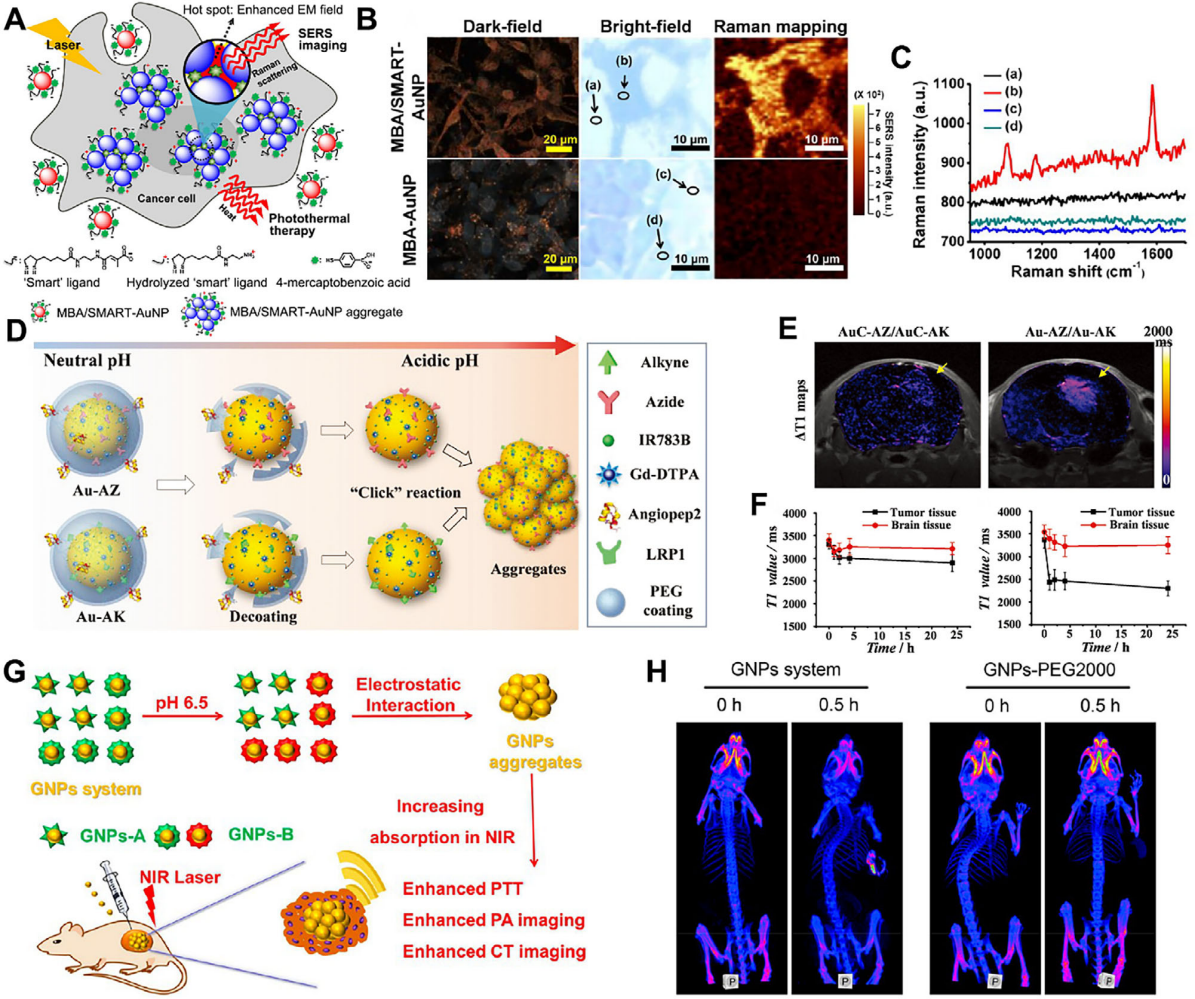
Stimuli‐responsive AuNPs aggregation for SERS imaging, MR imaging and CT imaging. (A) Schematic illustration of the action mechanism of MBA/SMART‐AuNP in tumor cell. Reproduced with permission [52]. Copyright 2013, American Chemical Society. (B) Dark‐field, bright‐field and Raman‐mapped images of tumor cells after treatment by MBA/SMART‐AuNPs or MBA‐AuNPs. Spots (a) and (c) represents background area outside of cells, and spots (b) and (d) represent area of cells. Reproduced with permission [52]. Copyright 2013, American Chemical Society. (C) Quantified Raman signals of different positions. Reproduced with permission [52]. Copyright 2013, American Chemical Society. (D) Schematic illustration of pH‐induced aggregation of Au–AZ and Au–AK trough cycloaddition reaction. Reproduced with permission [53]. Copyright 2017, Wiley. (E) Δ*T*1 maps indicated *T*1 value changes in the brain pixel by pixel at 24 h PI of the nanoprobe pair. Reproduced with permission [53]. Copyright 2017, Wiley. (F) Average *T*1 values of the tumor and contralateral normal brain tissue at 24 h PI of the pH‐inert (left) or pH‐responsive (right) nanoprobe pair. Reproduced with permission [53]. Copyright 2017, Wiley. (G) Schematic illustration of pH‐triggered aggregation of GNP system and enhanced imaging. Reproduced with permission [54]. Copyright 2019, American Chemical Society. (H) CT images of tumor‐bearing mice after treatment by GNP system and GNPs‐PEG2000. Reproduced with permission [54]. Copyright 2019, American Chemical Society.

SERS imaging is a non‐invasive vibration spectrum technology that utilizes strong Raman scattering at the surface of metallic nanostructures [58]. The LSPR of AuNPs aggregates can also lead to strong Raman scattering for SERS imaging [59]. When the laser irradiates the AuNPs aggregates, the photons of the incident light interact with the AuNPs aggregates to produce enhanced Raman scattering signals. These signals are further collected and analyzed by spectrometer for SERS imaging of tumor tissue [60]. SERS imaging holds great prospects in tumor diagnosis due to its ultra‐high sensitivity, unaffected stability and fingerprint‐like spectrum. Therefore, Kim et al. developed a pH‐responsive AuNPs aggregation system for SERS imaging. In the slightly acid tumor microenvironment, the AuNPs surface was simultaneously positively and negatively charged, which caused rapid AuNPs aggregation via electrostatic interaction (Figure 4A) [52]. Compared with the control group, the experimental group had a strong SERS signal due to the efficient accumulation of AuNPs (Figure 4B,C).

### 4.3 MR Imaging

MR imaging is a kind of detection technology based on the principle of nuclear spin [61]. MR imaging is widely used for tumor diagnosis [62]. The commonly used MR imaging contrast agent is gadolinium‐diethylenetriamine penta‐acetic acid (Gd^3+^‐DTPA), which has a unique electronic structure. The Gd^3+^‐DTPA will temporarily magnetized for MR imaging under an external magnetic field. However, the Gd^3+^‐DTPA has a short blood circulation time and therefore need repeated injections with high dosages [63], resulting in unexpected toxic side effects. The stimuli‐responsive AuNPs have long blood circulation and tumor accumulation time through change their sizes at the appropriate time under specific stimuli. Therefore, the Gd^3+^‐DTPA modified on stimuli‐responsive AuNPs will achieve accurate and safe MR imaging. Li et al. fabricated a pH‐responsive AuNPs system (Au‐AZ and Au‐AK) modified with azides and alkynes, which further protected by acid‐cleavable PEG shell [53]. During diagnosis, the Au‐AZ and Au‐AK were selectively enriched in brain tumor via low‐density lipoprotein‐receptor‐related protein‐1 mediated transcytosis. After that, the PEG protective shell decomposed to expose azides and alkynes under acidic conditions, leading to AuNPs aggregation and further activating MR imaging for guiding surgical resection (Figure 4D). The experimental results indicated that MR imaging could clearly show the outline of brain tumor (Figure 4E,F).

### 4.4 CT Imaging

CT imaging uses X‐ray scanning and then the scanned information is processed by computer to obtain final images [64]. The AuNPs aggregates have a high X‐ray absorption coefficient, which provides support for CT imaging. Liu et al. designed a smart pH‐responsive AuNPs system (GNPs‐A&B) in situ aggregation in the acidic tumor microenvironment for CT imaging of tumor [54]. GNPs‐A and GNPs‐B modified with Asp‐Asp‐Asp‐Asp‐Asp‐Cys and Lys‐Lys‐Lys‐Lys‐Lys‐Cys grafted to 2,3‐dimethylmaleic anhydride, respectively. The GNPs‐B could convert to positively charged in acidic conditions and further induced aggregation of GNPs‐A and GNPs‐B through electrostatic interactions, resulting in absorption peak shift to the NIR region for CT imaging (Figure 4G). As can be seen from Figure 4H, the CT imaging signal was obviously observed after treatment of GNPs‐A&B, while the control group did not observe the CT imaging signal.

## 5 Stimuli‐Responsive AuNPs Aggregation for Tumor Treatment

The tumor microenvironment is the soil for the survival of tumor cells, which promotes the occurrence, development and metastasis of tumors [65]. Compared with normal tissue, tumor tissue has unique properties, such as lower pH [66], overexpressed enzymes (alkaline phosphatase, furin, matrix metalloproteinase, and transglutaminase) [67], overexpressed cancer‐promoting RNA (TK1 mRNA, miR‐21 and miR‐155) [68] and overexpressed ions (Ca^2+^, Cu^2+^) [69]. In recent years, efforts have been made to manipulate AuNPs aggregation using these specific tumor microenvironment stimuli, which not only enhance the accumulation and retention of AuNPs in the tumor and transfer LSPR to the NIR region for effective PTT and radiotherapy but also manipulate or even reversing this specific tumor microenvironment for synergistic enhancement of tumor therapy. This part summarized in detail the use of specific tumor microenvironment stimuli to trigger AuNPs aggregation for tumor therapy (Table 2).

**TABLE 2:** Summary of main stimuli used AuNPs aggregation for tumor treatment.

Stimuli	Mechanism	Reference
pH	Electrostatic interactions (zwitterionic compounds); hydrogen bond (imidazole moiety); ion coordination bond (L‐cysteine)	[70, 71, 72, 73, 74, 75, 76, 77]
Enzyme	Peptide bond cleavage and formation	[78, 79, 80, 81, 82, 83, 84, 85]
RNA	Complementary base pairing	[86, 87]
Ions	Ion coordination bond	[88, 89, 90, 91, 92]

### 5.1 pH

Different tissues of the human body exhibit different pH levels. Because of the tumor tissue hypoxia, abnormal glycolysis, imbalance of ion channels, and vigorous metabolism lead to the pH of tumor tissue being lower than that of normal tissue at around 6.8, while the blood and normal tissues are maintained at 7.4 [93]. The mechanisms of pH‐responsive AuNPs aggregation include electrostatic interactions, hydrogen bonds or ionic coordination bonds of decorated pH‐sensitive surface molecules. Under acidic conditions, the pH‐responsive AuNPs will aggregate and prolong retention in tumor tissue for further therapy.

#### 5.1.1 Electrostatic Interactions

**FIGURE 5:**
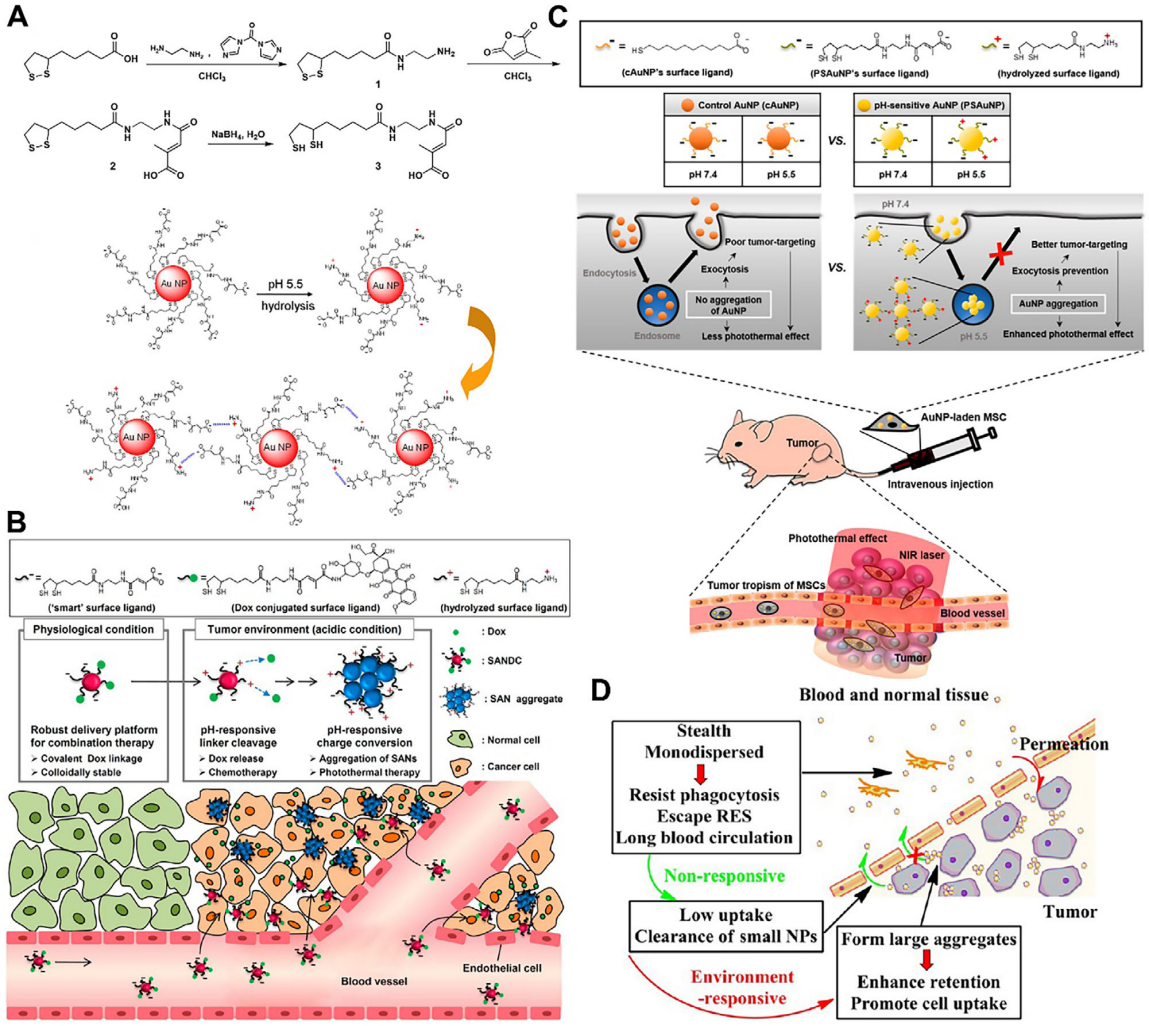
pH‐responsive AuNPs aggregation for tumor treatment. (A) The preparation process of compound **3** and illustration of pH‐induced aggregation of “smart” AuNPs via electrostatic attractions. Reproduced with permission [70]. Copyright 2009, American Chemical Society. (B) Schematic illustration of the working mechanism of SANDC. Reproduced with permission [71]. Copyright 2013, American Chemical Society. (C) Schematic illustration of the working mechanisms of the pH‐sensitive MSC‐PSAuNPs. Reproduced with permission [72]. Copyright 2015, American Chemical Society. (D) Schematic illustration of the working mechanisms of AuNPs aggregation in tumors that enhanced retention and cellular uptake. Reproduced with permission [73]. Copyright 2013, American Chemical Society.

The pH‐responsive molecules used for electrostatic interactions usually are zwitterionic compounds, such as citraconic amide moiety, 11‐mercaptoundecanoic acid, (10‐mercaptodecyl)trimethylammonium bromide and 2,3‐dimethylmaleic anhydride. The citraconic amides are rapidly converted to positively charged primary amines by hydrolysis under a slightly acidic pH, which could in turn react with the negatively charged carboxyl groups via electrostatic interactions to cause AuNPs aggregation. Based on citraconic amide moiety, Kim et al. first proposed the pH‐responsive AuNPs aggregation strategy for tumor‐specific PTT [70]. They ingeniously engineered this pH‐sensitive citraconic amide compound **3**, which had a negative electrical property and the electrical property could change to positive under acidic conditions. The synthetic route of compound **3** was shown in Figure 5A, a‐lipoic acid first reacted with ethylenediamine to from compound **1** via amidation reaction, which continued amidated with citric anhydride to obtain compound **2**. Then, the disulfide bond was cleaved under basic conditions to generate the pH‐sensitive sulfhydryl citraconic amide compound **3**, which was further modified to the surface of the AuNPs to obtain the final “smart” AuNPs via Au‐S bond. The prepared “smart” AuNPs were monodispersed in normal blood circulation. Once uptake by the tumor tissue through the EPR effect, the surface electrical properties of the “smart” AuNPs were partially reversed due to the acidic environment. Then, electrostatic interaction induced the AuNPs aggregation, causing the UV absorption peaks of AuNPs to shift to NIR region for tumor specific PTT. This article was the first to utilize pH‐inducing AuNPs aggregation via electrostatic interaction, which provided a reference for subsequent research. In order to further improve the efficiency of tumor treatment, Kim et al. grafted the chemotherapeutic drug doxorubicin (DOX) to this citraconic amide compound **3** and further constructed the new “smart” AuNPs (SAN) (Figure 5B) [71]. In the acidic tumor microenvironment, the surface ligands of SAN were partially hydrolyzed, which triggered charge transition and DOX release for SAN aggregation‐induced PTT and chemotherapy, respectively. This tumor‐specific PTT and chemotherapy were coordinated in space and time, so as to significantly inhibit tumor growth. As to increased tumor targeting of AuNPs, Kim et al. further encapsulated these pH‐sensitive AuNPs with mesenchymal stem cell (MSCs) membrane to construct MSC‐PSAuNPs (Figure 5C) [72]. MSCs had excellent tumor targeting ability, which endowed MSC‐PSAuNPs with the ability of tumor targeting, resulting in more nanoparticles entering into tumor tissue. Once uptake by tumor tissue, the MSC‐PSAuNPs were hydrolyzed under acidic conditions, resulting in a partial charge flipping. Then, electrostatic interactions induced MSC‐PSAuNPs aggregation, and further increased the local temperature upon NIR irradiation for PTT. Meanwhile, MSC‐PSAuNPs aggregates inhibited efflux and increased the residence time, enabling sustained treatment. They also fabricated a pH‐insensitive AuNPs as a control. Since the charge was not reversed under acidic conditions, the control group did not cause AuNPs aggregation. The results showed that the anti‐cancer effect of MSC‐PSAuNPs in the experimental group was significantly enhanced compared with the control group.

Different from using the citraconic amide moiety under acidic conditions to change the surface charge to induce AuNPs aggregation. Ji et al. designed the pH‐responsive mixed‐charge zwitterionic AuNPs, which were modified with the weak electrolytic 11‐mercaptoundecanoic acid and strong electrolytic (10‐mercaptodecyl)trimethylammonium bromide [73]. Under normal physiological conditions, due to zwitterionic properties, the AuNPs not eliminated from the circulation in the body. Once inside tumor tissues, the surface electrical properties of AuNPs were changed and exhibited rapid, ultrasensitive aggregation to acidity via electrostatic interactions, which generated large amounts of heat for PTT under NIR irradiation (Figure 5D).

**FIGURE 6:**
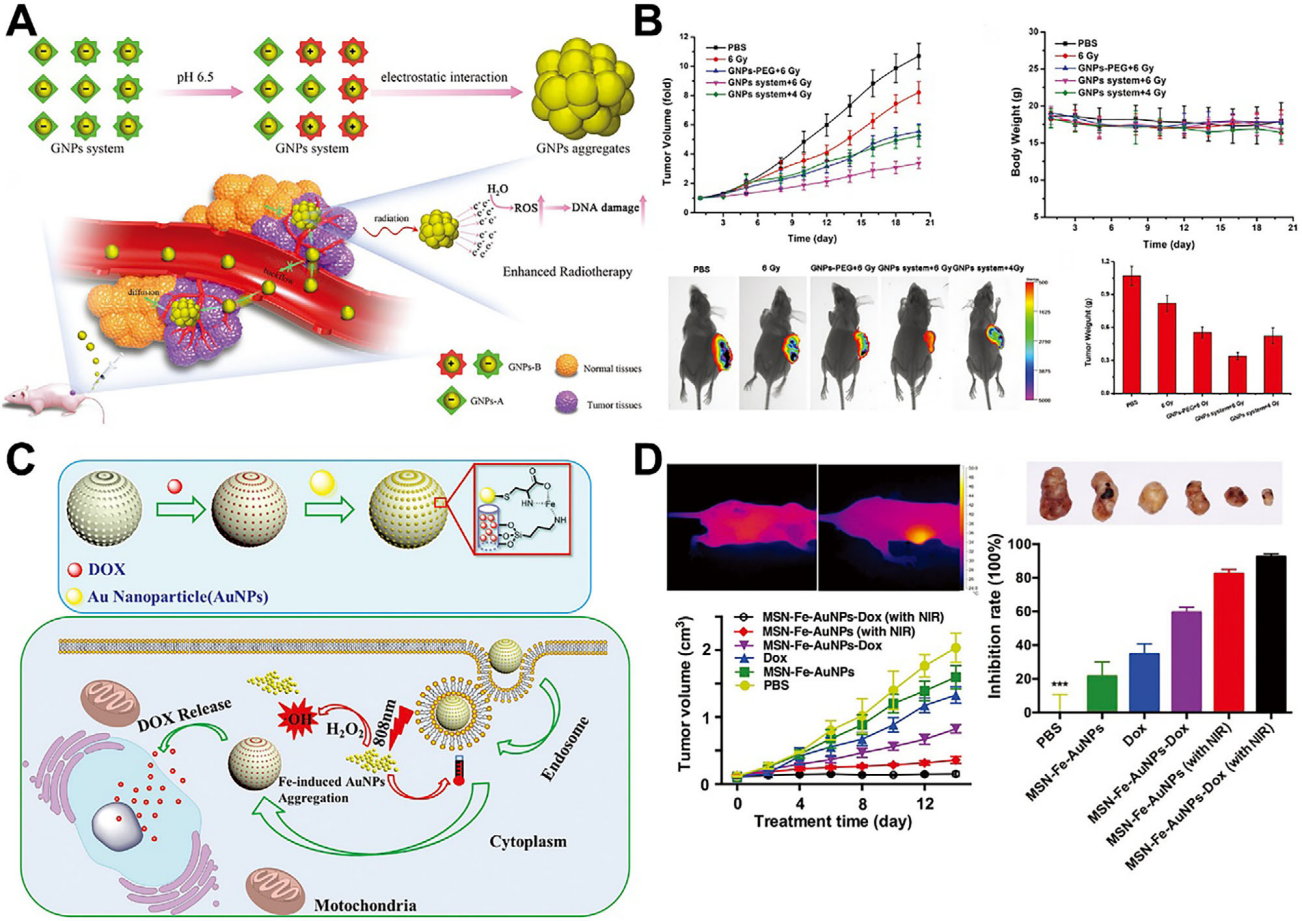
pH‐responsive AuNPs aggregation for tumor treatment. (A) Schematic illustration of pH‐induced aggregation of GNPs system and in vivo working mechanism of GNPs system for increased tumor retention and enhanced RT. Reproduced with permission [74]. Copyright 2019, Wiley. (B) Tumor volume, body weight, bioluminescent imaging and tumor weight in the mice after treatment with PBS, 6 Gy irradiation only, 6 Gy radiation after intravenous injection of GNPs‐PEG2000, and 6 or 4 Gy radiation after intravenous injection of GNPs system. Reproduced with permission [74]. Copyright 2019, Wiley. (C) Schematic illustration of fabrication and working mechanism of MSN‐Fe‐AuNPs. Reproduced with permission [77]. Copyright 2018, Wiley. (D) Photothermal images, tumor growth and tumor inhibition ratio of different treatments. Reproduced with permission [77]. Copyright 2018, Wiley.

In addition, the 2,3‐dimethylmaleic anhydride could also be rapidly converted to positively charged amines by hydrolysis at a mildly acidic condition, and in turn, react with the negatively charged carboxyl groups via electrostatic interactions to trigger AuNPs aggregation. Liu et al. designed a pair of AuNPs (GNPs‐A and GNPs‐B), which were modified with Asp‐Asp‐Asp‐Asp‐Asp‐Cys peptide and Lys‐Gly‐Gly‐Lys‐Gly‐Gly‐Lys peptide grafting 2,3‐dimethylmaleic anhydride, respectively [74]. The composition of the GNPs system and acid‐induced aggregation are shown in Figure 6A. The surface charges of GNPs‐A and GNPs‐B were both negatively charged, which monodisperse during normal blood circulation. After entering the tumor tissue, the surface charge of GNPs‐B was reversed from a negative charge to a positive charge due to hydrolysis of 2,3‐dimethylmaleic anhydride under acidic conditions, which led to opposite surface charges between GNPs‐A and GNPs‐B, resulting GNPs aggregation via electrostatic interactions. The GNP aggregates could generate more cytotoxic ROS under gamma radiation to increase DNA damage, thereby improving the efficiency of radiotherapy (Figure 6B). Subsequently, Gao et al. modified 2,3‐dimethylmaleic anhydride grafted polyethylene glycol on the AuNPs to fabricate an intelligent AuNPs‐D‐P‐DA system [75]. In the tumor acidic microenvironment, the negatively charged 2,3‐dimethylmaleic anhydride separated from the AuNPs system, causing the exposure of positively charged ends of PEG. Therefore, the AuNPs aggregates were formed in situ through electrostatic interaction for further antitumor therapy.

#### 5.1.2 Hydrogen Bond

The pH‐responsive molecule used for hydrogen bonds is imidazole moiety, which could rapidly protonate at a slightly acidic pH and form hydrogen bonds to trigger AuNPs aggregation. Liu et al. developed a pH‐responsive AuNPs (PMIZ‐AuNPs), which modified with PEG and zwitterionic imidazole moiety showed a negative charge [76]. Due to the electrostatic repulsion, the PMIZ‐AuNPs were monodisperse during blood circulation. At lower pH values, the imidazole groups protonated, resulting in the surface charge of the PMIZ‐AuNPs reversed and further triggered aggregation of PMIZ‐AuNPs via forming strong hydrogen bonds for further therapy.

#### 5.1.3 Ion Coordination Bond

The pH‐responsive molecule used for ion coordination bond is L‐cysteine, which could rapidly form an L‐cysteine‐Fe^2+^ coordination bond hydrogen bond to trigger AuNPs aggregation. Our group designed an intelligent pH‐responsive AuNPs (MSN‐Fe‐AuNPs) for trimodal tumor therapy based on ion coordination bond (Figure 6C) [77], This nanoplatform used MSN‐NH_2_ with DOX loading as the core and L‐cysteine‐modified gold nanoparticles (AuNPs‐Cys) as the shell, which connected via acid‐cleavable Fe^2+^ coordination bond. The AuNPs shell prevented the premature release of DOX. After application, MSN‐Fe‐AuNPs were enriched in tumor tissue and phagocytosed by tumor cells. Then, the low pH of lysosomes (pH = 5.0) could trigger the cleavage of Fe^2+^ coordination bonds between MSN‐NH_2_ and AuNPs, resulting in the detachment of AuNPs from the surface of MSN to further induce DOX release for chemotherapy. In addition, new Fe^2+^‐coordinated bonds between the exfoliated AuNPs were re‐formed to trigger AuNPs aggregation for PTT. At the same time, Fe^2+^ could catalyze the overexpressed H_2_O_2_ to generate a large number of cytotoxic hydroxyl radicals (·OH) for chemodynamic therapy [94]. The results showed that MSN‐Fe‐AuNPs significantly inhibited the growth of tumors (Figure 6D). Therefore, MSN‐Fe‐AuNPs nanoplatform realized the synergistic treatment of PTT, chemotherapy, and chemodynamic therapy based on the pH‐responsive AuNPs aggregation strategy.

### 5.2 Enzyme

**FIGURE 7:**
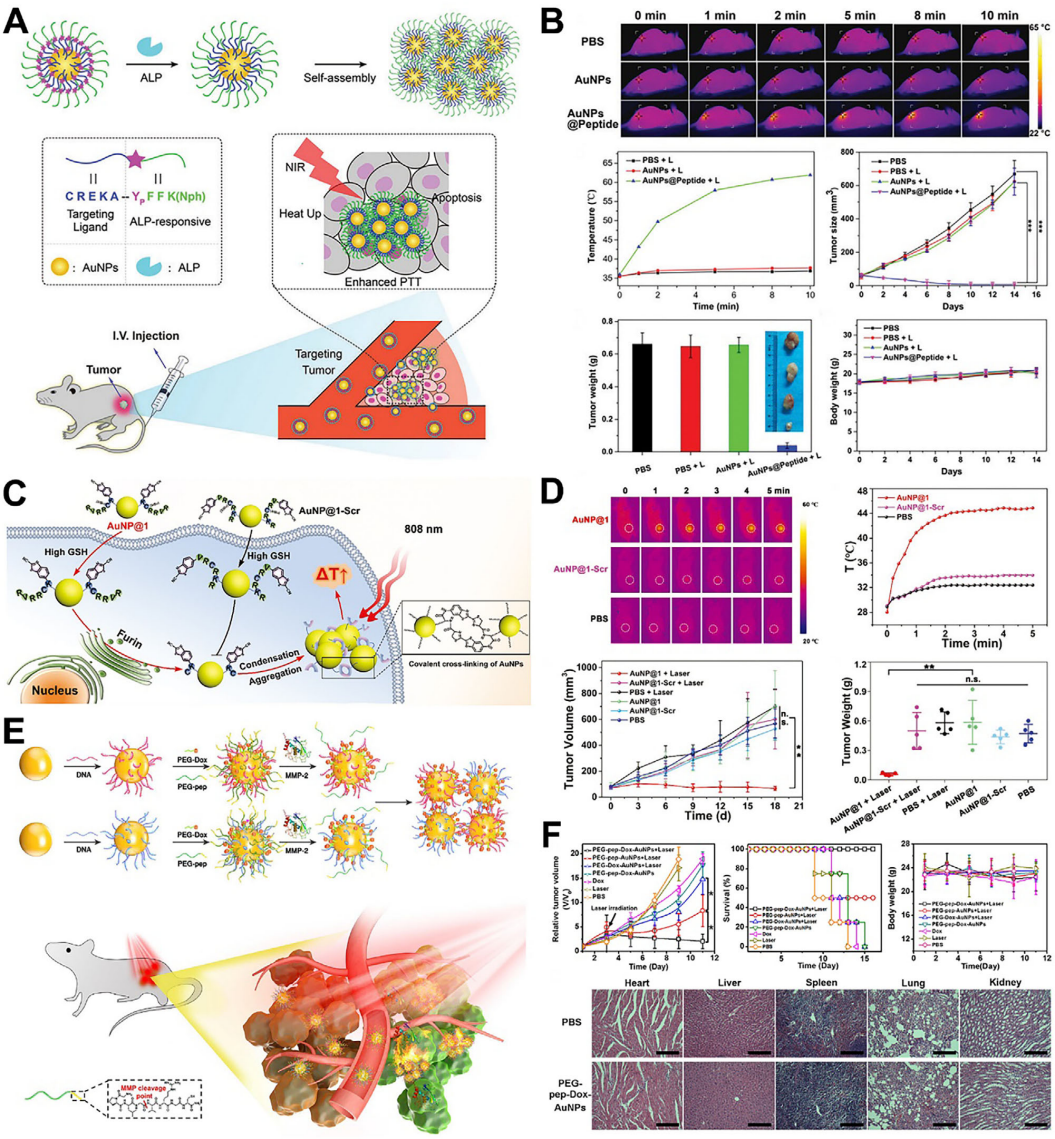
Enzyme‐responsive AuNPs aggregation for tumor treatment. (A) Schematic illustration of alkaline phosphatase‐induced aggregation of AuNPs@Peptide for enhanced PTT. Reproduced with permission [78]. Copyright 2018, Royal Society of Chemistry. (B) Thermal imaging, corresponding temperature change curves, tumor size, tumor weights, and body weight after treatments by PBS, PBS + L, AuNPs + L, and AuNPs@Peptide + L, L represents laser. Reproduced with permission [78]. Copyright 2018, Royal Society of Chemistry. (C) Schematic illustration of furin‐induced aggregation of AuNP@1 for PTT. Reproduced with permission [79]. Copyright 2020, Wiley. (D) Thermal imaging, temperature changes, tumor volume and tumor weights after treatments PBS, AuNP@1 and AuNP@1‐Scr with or without irradiation. Reproduced with permission [79]. Copyright 2020, Wiley. (E) Schematic illustration of MMP‐induced aggregation of AuNPs for enhanced PTT. Reproduced with permission [82]. Copyright 2019, Elsevier. (F) Tumor volume, survival curve, body weight and H&E‐stained tissue sections after treatments by PBS, 808 nm irradiation, free Dox, PEG‐pep‐Dox‐AuNPs, PEG‐Dox‐AuNPs + 808 nm irradiation, PEG‐pep‐AuNPs + 808 nm irradiation and PEG‐pep‐Dox‐AuNPs +808 nm irradiation. Reproduced with permission [82]. Copyright 2019, Elsevier.

Enzymes play an important role in the metabolism of organisms [95]. Tumor tissue contains various overexpressed enzymes, such as alkaline phosphatase, furin, legumain, matrix metalloproteinase (MMP), transglutaminase (TGase), etc. For the enzyme‐responsive AuNPs aggregation strategy, specific enzyme‐cleavable peptide ligands need to be modified on the surface of AuNPs. Wang et al. developed an alkaline phosphatase‐responsive AuNPs (AuNPs@Peptide) [78]. The used AuNPs surface ligand was CREKA‐YPFFK (Nph), which consisted of CREKA and YPFFK (Nph) responsible for tumor targeting and alkaline phosphatase‐responsive bond breaking, respectively (Figure 7A). During therapy, AuNPs@Peptide could accumulate in tumor tissue due to the CREKA ligands‐induced tumor targeting. Then, the phosphate group of the YPFFK (Nph) fragment was cleaved by alkaline phosphatase, resulting in the AuNPs aggregation via hydrogen bonds. Therefore, aggregated AuNPs served as excellent photothermal agents for PTT. Meanwhile, aggregation prolong the residence time for long‐term therapy. The results indicated that AuNPs@Peptide significantly inhibited the tumors (Figure 7B).

Furin is a type of trans‐Golgi protein convertase upregulated in cancer cells, which is essential for tumor proliferation. Based on this protein, Wang et al. designed a furin‐responsive AuNPs (AuNP@1), which was modified with Ac‐Arg‐Val‐Arg‐Arg‐Cys(StBu)‐Lys‐CBT (Figure 7C) [79]. Once uptake by tumor cell, the overexpressed furin and glutathione could respectively split the furin‐responsive peptide and reduce the disulfide bond to expose the 1,2‐aminothiol group of Cys. Then, the 2‐cyanobenzothiazole (CBT) and Cys undergo a condensation reaction to induce the AuNP@1 aggregation for PTT (Figure 7D). In addition, Gao et al. also fabricated a furin‐responsive AuNPs system (AuNPs‐D&H‐R&C), which was composed of RVRRCK peptide‐modified AuNPs and 2‐cyano‐6‐aminobenzothiazole‐modified AuNPs, both co‐loaded with doxorubicin and hydroxychloroquine [80]. The overexpressed furin in tumor tissue could specifically cleave the RVRRCK peptide to expose 1,2‐thiolamino group, which further reacted with cyano group on 2‐cyano‐6‐aminobenzothiazole‐polyethylene through a click cycloaddition, causing in situ aggregation of AuNPs to enhanced drug accumulation within tumors. Meanwhile, doxorubicin and hydroxychloroquine synergistically mediated chemotherapy and macrophage reprogramming for treatment of breast cancer. Besides, Gao et al. further designed a legumain‐responsive AuNPs system (D&H‐A‐A&C) [81]. The D&H‐A‐A&C consisted of two kinds of AuNPs, which were Ala‐Ala‐Asn‐Cys‐Lys modified AuNPs and 2‐cyano‐6‐amino‐benzothiazole modified AuNPs, co‐loaded with doxorubicin and hydroxychloroquine. The D&H‐A‐A&C could passively target the glioma site via the EPR effect and trigger AuNPs in situ aggregation in response to the overexpressed legumain, leading to enhanced drug accumulation for improved glioma treatment.

Subsequently, Nie et al. designed MMP‐responsive AuNPs to realize the integration of PAI, PTT and chemotherapy [82]. This AuNPs was modified with three functional molecules, which were complementary paired DNA strands, thermally labile 4'4‐azobis (4‐cyanovaleric acid)‐linked DOX and MMP‐cleavable peptide‐PEG, respectively (Figure 7E). On reaching the overexpressed‐MMP tumor tissue, the peptide‐PEG was cleaved to expose complementary DNA strands. Driven by DNA hybridization, the AuNPs rapidly aggregated for PAI and PTT. With the local temperature raised, DOX was released to synergistically enhance tumor treatment efficiency (Figure 7F).

**FIGURE 8:**
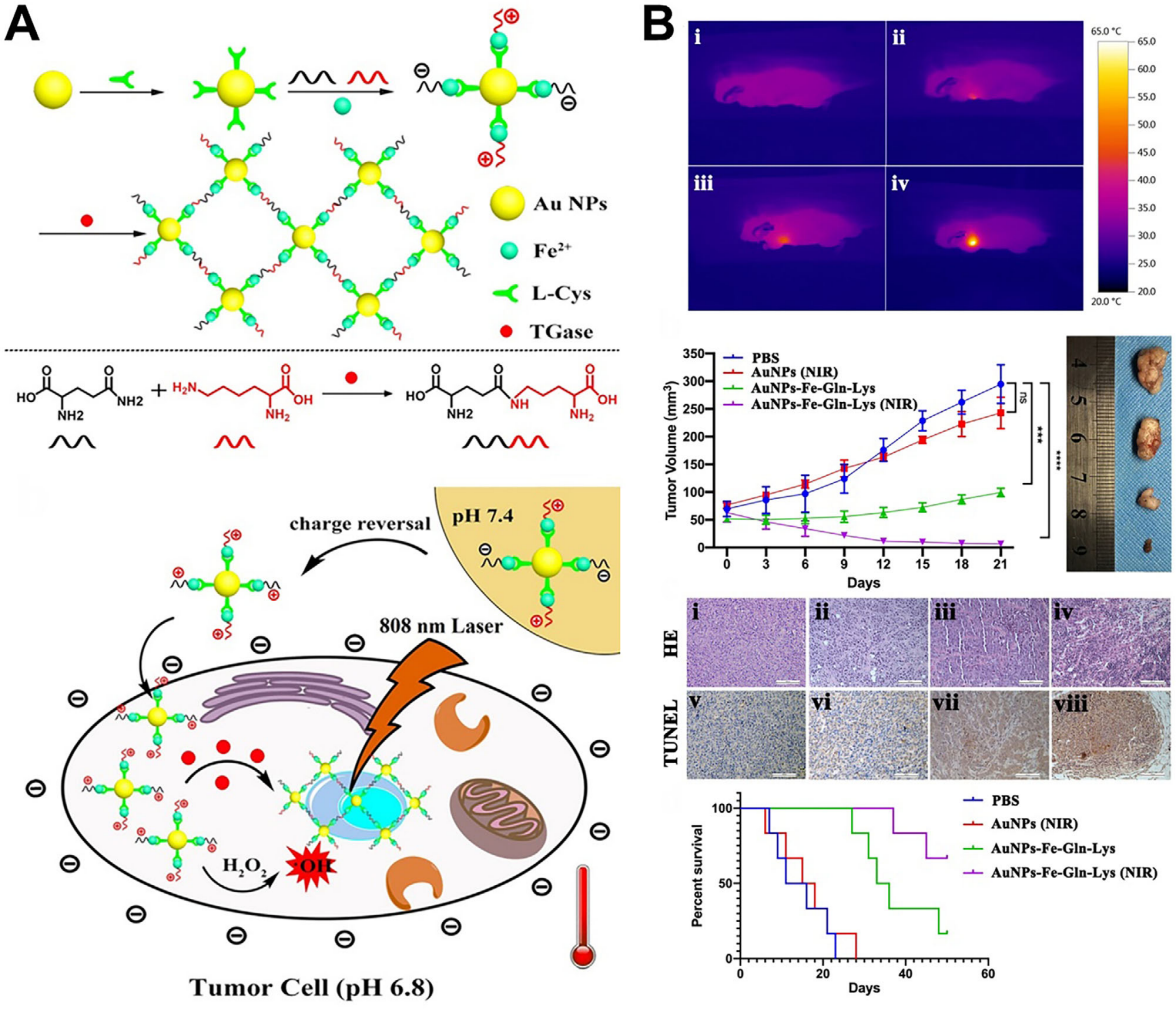
TGase‐responsive AuNPs aggregation for tumor treatment. (A) Schematic illustration of the fabrication and TGase‐triggered aggregation of AuNPs‐Fe‐Glu‐Lys for enhanced PTT. Reproduced with permission [83]. Copyright 2019, American Chemical Society. (B) Photothermal images, tumor volume, H&E stained tissue sections and survival curves of mice after treatment by PBS, AuNPs with NIR irradiation and AuNPs‐Fe‐Glu‐Lys without or with NIR irradiation. Reproduced with permission [83]. Copyright 2019, American Chemical Society.

**FIGURE 9:**
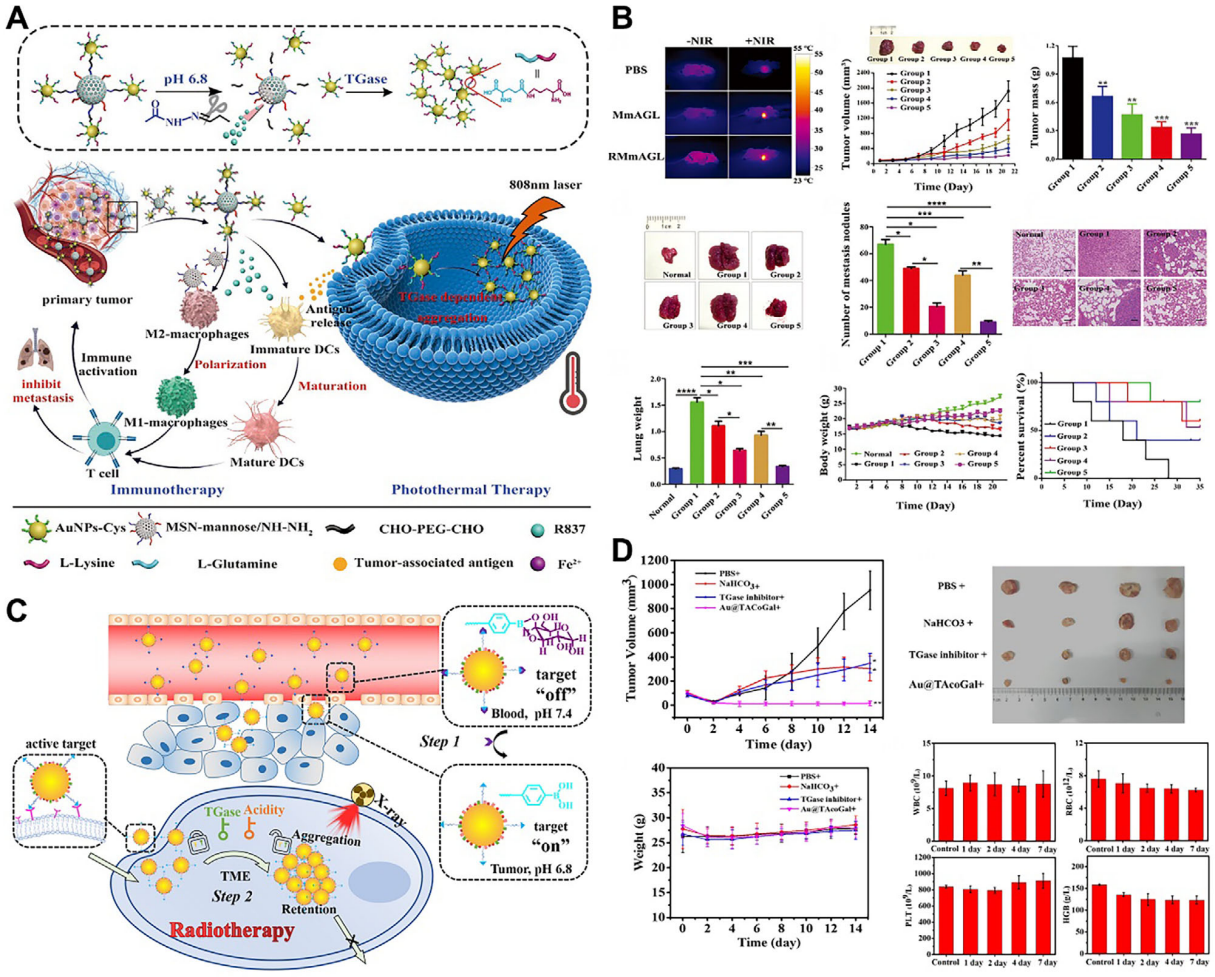
TGase‐responsive AuNPs aggregation for tumor treatment. (A) Schematic illustration of the working mechanism of pH‐TGase‐NIR multi‐responsive immune‐adjuvant RMmAGL. Reproduced with permission [84]. Copyright 2023, Wiley. (B) Photothermal images, tumor volume, tumor mass, lung photographs, number of metastatic nodules, H&E staining of lung samples, lung weight, body weight, and survival curves of mice after different treatments. Group 1: PBS; Group 2: MmAGL; Group 3: RMmAGL; Group 4: MmAGL with NIR irradiation; Group 5: RMmAGL with NIR irradiation. Reproduced with permission [84]. Copyright 2023, Wiley. (C) Schematic illustration of the working mechanism of Au@TAcoGal. Reproduced with permission [85]. Copyright 2021, American Chemical Society. (D) Tumor volume, tumor photographs, body weights and blood tests after different treatments. Reproduced with permission [85]. Copyright 2021, American Chemical Society.

In addition to cleaving specific peptide ligands for exposing functional molecules to induce AuNPs aggregation, enzymes could also induce peptide bond formation to trigger aggregation. TGase was overexpressed in tumor cells and catalyzed the formation of peptide bonds between glutamate (Glu) and lysine (Lys). Inspired by these ideas, our group fabricated TGase‐responsive nanoparticles (AuNPs‐Fe‐Glu‐Lys), which exhibited excellent tumor selectivity, photothermal properties and catalytic medicine (Figure 8A) [83]. Glu and Lys were co‐modified to the surface of AuNPs through Fe^2+^ coordination bonds, making them have a pH‐sensitive zwitterionic surface. The AuNPs‐Fe‐Glu‐Lys were electrically neutral in the normal physiological environment (pH = 7.4). Under the acidic condition of tumor tissue (pH = 6.8), the surface electricity converted to a positive charge, which promoted the endocytosis of tumor cells. After that, TGase catalyzed the formation of peptide bonds between Glu and Lys to induce the AuNPs aggregation for PTT. In addition, Fe^2+^ further catalyzed the overexpressed H_2_O_2_ to generate a large amount of cytotoxic ·OH through the Fenton reaction, causing synergistic tumor damage (Figure 8B). Considering the metastasis and recurrence of malignant tumors, our group further combined the TGase dependent PTT with immunotherapy to construct RMmAGL nanoparticles, which consisted of a core of mannose‐modification mesoporous silica nanoparticles loaded with the TLR7 agonist R837 (R837@MSN‐mannose) connected via hydrazone bonds to Glu and Lys co‐modified AuNPs (AuNPs‐Glu/Lys) (Figure 9A) [84]. After application, the acidic tumor microenvironment cleaved the hydrazone bonds to expose AuNPs‐Glu/Lys, which further uptake by tumor cells. Then, TGase catalyzed peptide bond formation between Glu and Lys to induce AuNPs aggregation, loading to tumor‐specific PTT. The R837 along with the tumor‐associated antigens generated by PTT could mature dendritic cells and further activate T cells for immunotherapy. In addition, MSN‐mannose induced the polarization of macrophages from M2 to M1 for enhancing immunotherapy. The results showed that RMmAGL could not only inhibit primary tumor but also inhibit tumor metastasis (Figure 9B).

Wang et al. also designed a smart Au@TAcoGal nanoplatform using TGase [85]. They simultaneously modified with hydrophobic p‐mercaptobenzoic acid, Lys, Glu, and tumor targeting molecule phenylboronic acid onto AuNPs. Then, galactose was combined with phenylboronic acid to shield its targeting effect, and restored in the acidic tumor microenvironment. After entering tumor cells, TGase immediately catalyzed the formation of a peptide bond between Lys and Glu to induce AuNPs aggregation for enhanced radiotherapy (Figure 9C,D).

### 5.3 RNA

**FIGURE 10:**
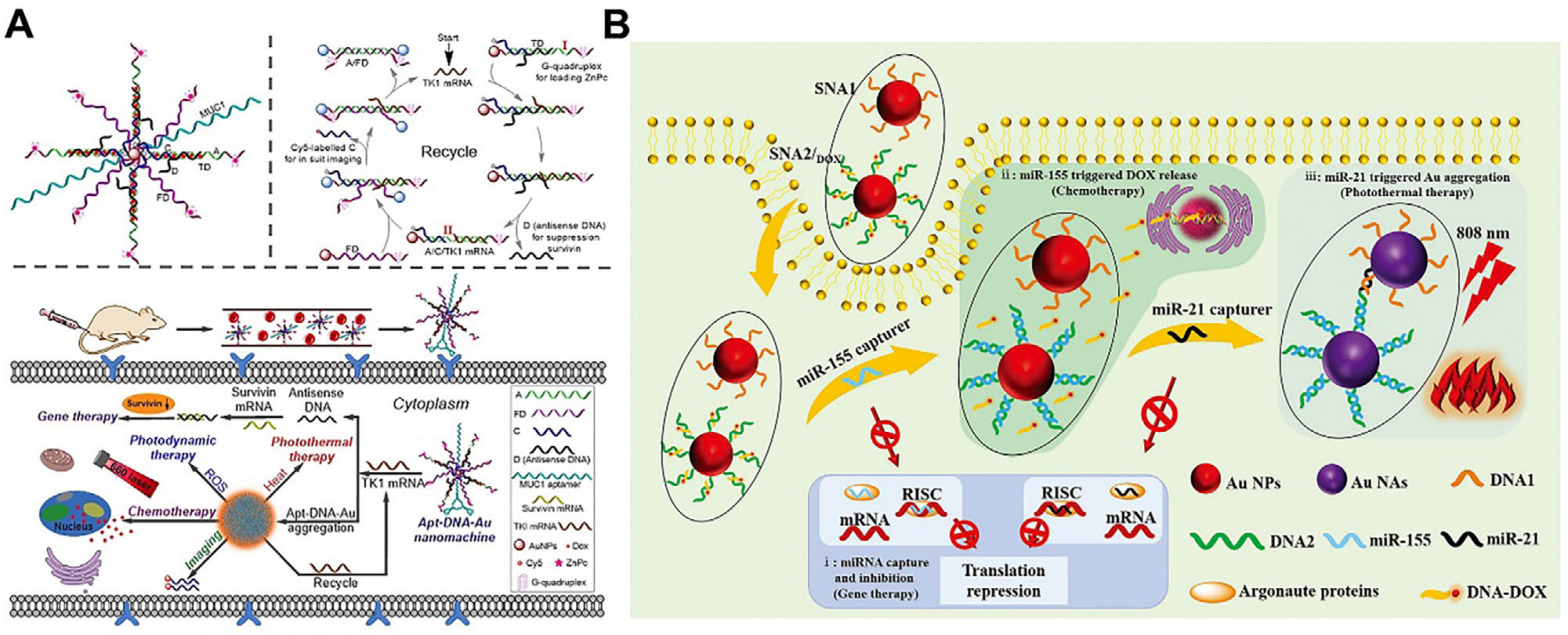
RNA‐responsive AuNPs aggregation for tumor treatment. (A) Schematic illustration of preparation and RNA‐induced aggregation of Apt‐DNA‐Au for multimodal synergistic cancer therapy. Reproduced with permission [86]. Copyright 2021, Wiley. (B) Schematic of the therapeutic mechanism of miR‐21 induced aggregation of SNAs for tumor‐specific genetic therapy, chemotherapy, and PTT. Reproduced with permission [87]. Copyright 2022, Wiley.

RNA‐responsive AuNPs aggregation for tumor therapy was achieved by complementary base pairing [96]. Zhu et al. first applied RNA‐responsive AuNPs aggregation for in vivo cancer therapy [86]. They fabricated a tumor‐associated TK1 mRNA‐responsive AuNPs (Apt‐DNA‐Au). After the specific internalization of Apt‐DNA‐Au into tumor cells, TK1 mRNA activated the Apt‐DNA‐Au through DNA strand displacement reaction, resulting in Apt‐DNA‐Au aggregation, the release of fluorophore and antisense DNA, which was used for PTT, cancer imaging and gene therapy, respectively. Meanwhile, DOX was released for chemotherapy and the loaded photosensitizer generated ROS under laser irradiation for photodynamic therapy. This nanoplatform was based on TK1 mRNA‐triggered Apt‐DNA‐Au aggregation and achieved multimodal integrated therapy (Figure 10A).

Subsequently, our group designed a smart spherical nucleic acid (SNA) based on oncogenic overexpressed microRNAs (miR‐21/miR‐155) [87]. This SNA was consisted of AuNPs as the core and DOX‐grafted antisense oligonucleotide as the shell. Antisense oligonucleotides could capture overexpressed miR‐21/miR‐155 causing SNA aggregation for PTT. In addition, the capture process reduced the level of miR‐21/miR‐155 for gene therapy. Meanwhile, DOX was released for chemotherapy. Our designed SNA not only induced aggregation by oncogenic microRNAs, but also reverse oncogenic tumor microenvironment for better cancer therapy (Figure 10B).

### 5.4 Ions

**FIGURE 11:**
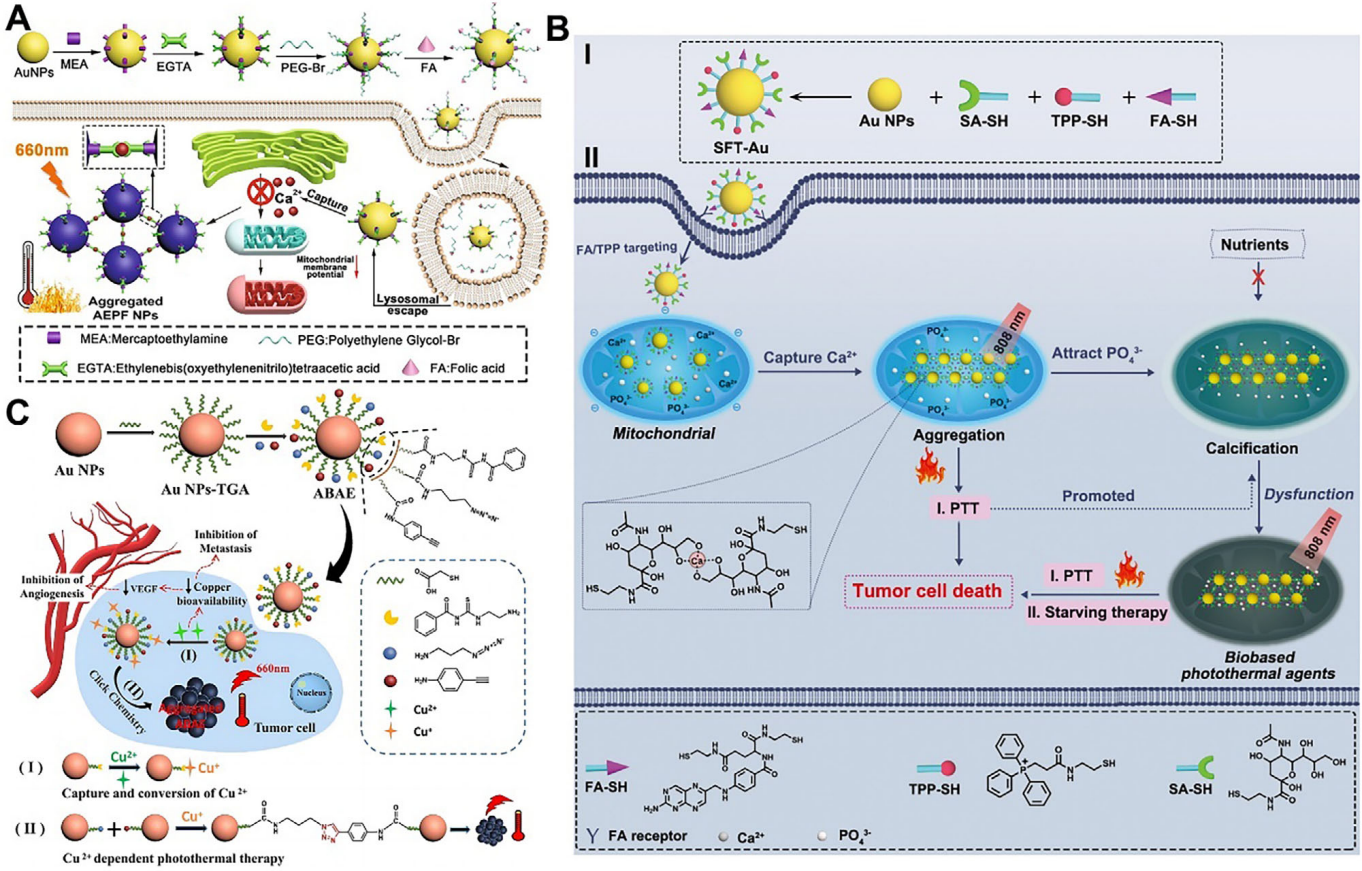
Ions‐responsive AuNPs aggregation for tumor treatment. (A) Schematic illustration of synthesis and Ca^2+^‐induced aggregation of AEPF NPs for tumor‐specific PTT. Reproduced with permission [88]. Copyright 2022, Elsevier. (B) Schematic illustration of synthesis and Ca^2+^‐triggered aggregation of SFT‐Au in tumor mitochondria for precise PTT and mitochondrial calcification‐mediated starving therapy. Reproduced with permission [89]. Copyright 2023, Wiley. (C) Schematic representation of synthesis and Cu^2+^‐induced aggregation of ABAE NPs for accurate PTT and copper capture therapy. Reproduced with permission [91]. Copyright 2023, Elsevier.

The tumor microenvironment contained many overexpressed ions (such as Ca^2+^ and Cu^2+^), which played an important role in tumor proliferation and metastasis [97]. Therefore, on‐demand regulation of this ion level may effectively inhibit tumors. Inspired by these ideas, our group designed a smart nanoplatform (AEPF NPs), AEPF NPs could capture Ca^2+^ and further induce its aggregation for PTT [88]. The preparation and function of AEPF NPs are shown in Figure 11A, AEPF NPs were composed of AuNPs as the core, Ca^2+^ capture agent EGTA as the shell, and PEG‐FA as the corona, where the EGTA and PEG‐FA were connected via esterase‐cleavable ester bonds. After AEPF NPs enriched in tumor cells by folic acid‐induced tumor targeting, PEG‐FA was dissociated to expose EGTA by overexpressed esterase, which further captured Ca^2+^ to trigger AuNPs aggregation for PTT. At the same time, the level of Ca^2+^ was decreased, which reversed the tumor growth microenvironment and inhibited the growth of tumor cells. In response to the limited Ca^2+^ concentration, uncontrolled and slow calcification in calcification‐based starvation therapy, our group fabricated a sialic acid, folic acid and triphenylphosphine co‐modified AuNPs (SFT‐Au). SFT‐Au could enter tumor mitochondria through folic acid‐induced tumor targeting and triphenylphosphine‐induced mitochondria targeting [89]. Then, the sialic acid fragment captured Ca^2+^ to cause AuNPs aggregation, this process not only accumulated Ca^2+^ to trigger the calcification of mitochondria, but in situ formed a photothermal agent for PTT and promoted the calcification, causing a block energy supply for starving therapy and photothermal damage of tumor cells to improve the efficiency of treatment (Figure 11B). In addition, Ca^2+^ is a vital ion for the normal operation of mitochondria and Golgi apparatus in tumor cells, the Ca^2+^ interference would be an effective strategy to disrupt these organelles for tumor treatment. Therefore, our group also fabricated a Ca2+‐responsive gold‐silicon nanoplatform (BMA_EF_) [90]. The BMA_EF_ could capture Ca^2+^ between mitochondria, endoplasmic reticulum and Golgi apparatus to block Ca^2+^ conduction, which not only induced mitochondria and Golgi apparatus dysfunction, but caused AuNPs aggregation to in situ generate photothermal agent for tumor‐specific PTT and PTT assisted immunotherapy.

Considering the importance of Cu^2+^ in tumor proliferation and metastasis, our group further developed a Cu^2+^‐responsive nanoplatform (ABAE). ABAE consisted of 3‐azidopropylamine, 4‐ethynylaniline and *N*‐aminoethyl‐*N*’ benzoylthiourea (BTU) co‐modified on its surface [91]. The BTU could capture Cu^2+^ to decrease the Cu^2+^ level to inhibit metastasis. At the same time, the Cu^2+^ became Cu^+^ through BTU to catalyze the click reaction between azido and alkynyl, which further triggered AuNPs aggregation for tumor‐specific PTT (Figure 11C). Although these ion‐dependent AuNPs opened a window for precise PTT, these therapeutic processes were not visible. It was a great challenge to perform the NIR irradiation at the proper time point and location to achieve optimal PTT. To solve this problem, our group further fabricated a Cu^2+^‐ responsive AuNPs (AuNTF), which was modified with aggregation‐induced‐emission (AIE) molecule [92]. The AuNTF could capture the over‐expressed Cu^2+^ to induce AuNTF aggregation and would generate the photothermal agent for PTT and simultaneously perform Cu2+‐mediated AIE imaging. The AIE imaging not only monitored the whole therapeutic process in real‐time, but was used to guide the PTT for optimal therapeutic efficiency.

## 6 Conclusions and Perspectives

In summary, we have systematically reviewed the function of stimuli‐responsive aggregation of AuNPs and its application in tumor diagnosis and treatment. The stimuli‐responsive AuNPs can meet the unique needs of tumor diagnosis and treatment at different stages through structural change, which has exhibited long blood circulation and enhanced tumor accumulation. Moreover, the stimuli‐responsive AuNPs have unique LSPR in the NIR region and a high X‐ray absorption coefficient can be used for tumor imaging, PTT and radiotherapy. However, despite the recent results of research on stimuli‐responsive AuNPs aggregation in tumor diagnosis and therapy are indeed encouraging, there are still many challenges that should be more attention in the future.

(1) The modification of AuNPs is relatively complicated, the ratio and purity of different components should be paid more attention. (2) As a general challenge in tumor therapy, heterogeneity among patients complicates the selection of AuNPs, and more attentions are needed to overcome this difficulty. (3) Tumor selectivity of gold nanoparticles needs to be further improved to achieve tumor‐specific aggregation and to avoid enrichment in normal tissues. (4) The complex blood circulation in the body may challenge the intended properties of some AuNPs. (5) Our knowledge of the biological fate of AuNPs aggregates is relatively limited, and systematic toxicological studies should be further strengthened. Considering the successful application of stimuli‐responsive AuNPs for tumor imaging and therapy, the future development of strategies to address these challenges and achieve more accurate and effective integration of cancer diagnosis and treatment is very promising. We believe that the stimuli‐responsive AuNPs system will eventually be applied to real clinical treatments.

## Conflicts of Interest

The authors declare no conflicts of interest.
